# Response of Sclerostin and Bone Turnover Markers to High Intensity Interval Exercise in Young Women: Does Impact Matter?

**DOI:** 10.1155/2018/4864952

**Published:** 2018-11-01

**Authors:** R. Kouvelioti, N. Kurgan, B. Falk, W. E. Ward, A. R. Josse, P. Klentrou

**Affiliations:** ^1^Department of Kinesiology, Faculty of Applied Health Sciences, Brock University, 1812 Sir Isaac Brock Way, St. Catharines, Ontario, Canada; ^2^Centre for Bone and Muscle Health, Faculty of Applied Health Sciences, Brock University, 1812 Sir Isaac Brock Way, St. Catharines, Ontario, Canada

## Abstract

This study examined potential exercise-induced changes in sclerostin and in bone turnover markers in young women following two modes of high intensity interval exercise that involve impact (running) or no-impact (cycling). Healthy, recreationally active, females (n=20; 22.5±2.7 years) performed two exercise trials in random order: high intensity interval running (HIIR) on a treadmill and high intensity interval cycling (HIIC) on a cycle ergometer. Trials consisted of eight 1 min running or cycling intervals at ≥90% of maximal heart rate, separated by 1 min passive recovery intervals. Blood samples were collected at rest (pre-exercise) and 5 min, 1h, 24h, and 48h following each exercise trial. Serum was analyzed for sclerostin, cross linked telopeptide of type I collagen (CTXI), and procollagen type I amino-terminal propeptide (PINP). A significant time effect was found for sclerostin, which increased from pre-exercise to 5 min after exercise in both trials (100.2 to 131.6 pg/ml in HIIR; 102.3 to 135.8 pg/ml in HIIC, p<0.001) and returned to baseline levels by 1h, with no difference between exercise modes and no exercise mode-by-time interaction. CTXI did not significantly change following either trial. PINP showed an overall time effect following HIIR, but none of the post hoc pairwise comparisons were statistically significant. In young women, a single bout of high intensity exercise induces an increase in serum sclerostin, irrespective of exercise mode (impact versus no-impact), but this response is not accompanied by a response in either bone formation or resorption markers.

## 1. Introduction

High-impact exercise such as running exerts larger mechanical loading on the skeleton compared to low- and no-impact exercise such as cycling [1]. In both cases, muscle contraction forces are applied to the bone, but in high-impact exercise (e.g., running), added loading is applied from the ground reaction forces. Thus, high-impact activities are most likely to have beneficial effects on bone metabolism and health [2, 3]. Numerous studies have reported higher bone mineral density in athletes of high-impact activities (e.g., gymnastics, basketball) than athletes of low- or no-impact activities (e.g., swimming, cycling) [2, 3]. However, it is less clear whether low-impact exercise imparts sufficient strain in the bone to stimulate any beneficial effect. This is essential information in designing interventions for people who need osteogenic activities but cannot tolerate the impact of ground reaction forces (e.g., individuals with osteoporosis) and those who choose not to engage in high-impact exercise.

Of particular importance in examining the role of impact (i.e., ground reaction forces), applied through exercise on bone, is the protein sclerostin due to its bone specific action. Sclerostin is a key osteokine that downregulates bone formation by inhibition of the canonical Wnt/*β*-catenin signaling pathway [4, 5] and promotes bone resorption by increasing receptor activator of nuclear factor *κ*B ligand (RANKL) secretion from osteocytes [6–8]. Sclerostin is mainly produced by osteocytes in response to mechanical unloading [5, 9]. Specifically, gene and protein expression of sclerostin decrease in response to mechanical loading both* in vitro* [10] and* in vivo* [11] while mechanical unloading has shown to increase gene and protein expression of sclerostin [11]. In humans, few studies have examined the response of sclerostin to exercise-induced mechanical loading, reporting disparate results. In particular, we have shown that plyometric exercise leads to an immediate increase in serum sclerostin levels in men [12], but not in prepubertal boys and girls [13]. Likewise, sclerostin appears to increase within 5 min following low intensity running in young women [14], as well as following a high intensity, long-duration (3-week) stage race in male cyclists [15]. Thus, sclerostin's response to exercise is not yet clear and may be related to mode, duration and intensity of exercise. Importantly, the differential acute response of sclerostin to impact versus no-impact exercise has not been examined and could provide insight on the specificity of the bone response to different modes of exercises in the short-term.

Furthermore, sclerostin has been associated with both bone formation and resorption markers in humans [16–18], but these findings are not consistent and differ depending on the age of the participants. For example, sclerostin has been shown to be negatively correlated with bone formation markers such as alkaline phosphatase (BAP) and procollagen type I amino-terminal propeptide (PINP), as well as with bone resorption markers such as cross linked telopeptide of type I collagen (CTXI) in older men and postmenopausal women [16–18] while it was found to correlate positively with both CTXI and BAP in premenopausal women [16]. In addition, these associations were studied only at rest and not in response to exercise. Examining whether exercise-induced changes in sclerostin are accompanied, or followed, by changes in bone turnover markers is important for expanding our understanding of the role of sclerostin in terms of the adaptive effect of bone to exercise.

Finally, there are numerous studies examining the effect of various modes of exercise (impact or non-impact) on the acute response of bone turnover markers, reporting contradictory results [19–21]. One explanation may be that these studies utilized different types and modes of exercise, rendering exercise type, or mode comparison problematic. Thus, the role of impact on the specificity of the bone turnover response to different types of exercise is unclear. Only one study to date has compared the exercise response of bone formation (osteocalcin, BAP) and resorption (NTX) markers between moderate intensity impact (jogging) and no-impact exercise (water aerobics) in the same individuals (young women), reporting no significant time or exercise mode effect in any of the markers up to 24h following the exercise [22]. Differences in the acute response of bone turnover markers between impact and no-impact exercise at different time points, and beyond 24h after exercise, can provide insight into the processes that may be driving the short and long-term bone adaptations to exercise.

This study compared the response of sclerostin to two modes of high intensity exercise (impact, running versus no-impact, cycling) in young women and examined whether potential exercise-induced changes in sclerostin are accompanied by changes in bone resorption (CTXI) and formation (PINP) markers. Based on the few previous human studies, it was hypothesized that both high intensity interval exercise modes will induce a significant increase in sclerostin and bone turnover markers immediately after exercise, all returning to baseline by 48h after exercise, and with larger changes expected following the running than the cycling, due to the higher impact applied on the skeleton.

## 2. Methods

### 2.1. Participants

Twenty (18 Caucasians and 2 Asians) females, 18-28 years old, were invited to participate in this study based on the following inclusion criteria: healthy, recreationally active (i.e., exercising 2 to 5 times per week), free of injuries or chronic conditions (e.g., ACL or knee/hip/lower back injuries, arthritis, and neuromuscular diseases), having no fracture in the last year, nonsmokers, and not taking any medication or dietary supplements affecting bone health (e.g., protein, vitamin D, and calcium). All participants agreed to participate in this study by signing a consent form. The study was conducted in accordance with the Declaration of Helsinki and received ethics approval from our institutional Research Ethics Board.

### 2.2. Study Design and Procedures

This study used a crossover, within-subject design, where each participant performed two high intensity exercise trials, in random order: a high intensity interval running (HIIR) trial on the treadmill and a high intensity interval cycling (HIIC) trial on a cycle ergometer. All participants visited the lab twice prior to the high intensity exercise trials. During these two preliminary visits, scheduled 1-3 days apart, participants were informed about the study and signed the consent form, completed the medical history questionnaire that was used to verify the inclusion criteria, and had their anthropometric and body composition measurements taken. Subsequently, they performed two incremental exercise tests to exhaustion (cycling on a cycle ergometer and running on a treadmill) in random order (one exercise test in each visit), which were used to determine the maximal workload. Maximal workload was determined by volitional fatigue, when participants could no longer continue pedaling or running. The maximal speed and incline (running) and watts (cycling) at the point of exhaustion as well as heart rate and perceived exertion by Borg scale were recorded.

**Figure 1:**
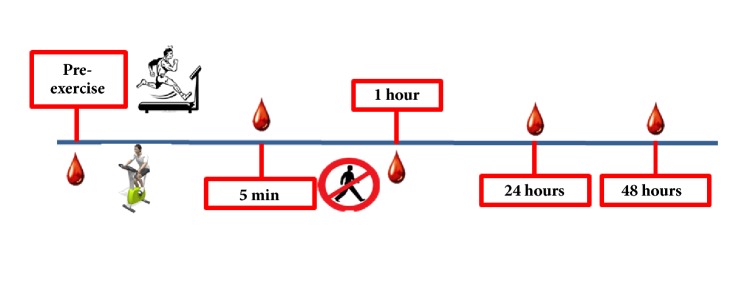
High intensity interval exercise trials (HIIR and HIIC) procedures.

In the subsequent visits, participants performed the two high intensity exercise trials (HIIR and HIIC) in random order. During both trials, 5 blood samples were collected: pre-exercise, and 5 min, 1h, 24h, and 48h after exercise (Figure 1). All visits were scheduled in the morning between 1000 and 1200 hours to control for diurnal variation in the biochemical markers. For consistency, before every visit to the laboratory, participants were instructed to consume the same standardized breakfast at home, which included one slice of whole grain bread with butter/margarine or peanut butter, one glass of 2% milk or one cup of 2% fat yogurt, one banana or apple, and one cup of coffee or tea. Likewise, they were instructed not to eat or drink (except water) for about 2 hours after their breakfast and prior to their laboratory visits.

Prior to all visits to the laboratory, participants were asked to avoid alcohol and exercise for 24h before their two preliminary visits, and 48h before and following the high intensity interval exercise trials. All the visits took place within three consecutive weeks following the week of menstruation. All participants were on birth control; 17 were on oral contraceptives with downregulated hormonal profiles, 2 used a hormonal intrauterine device and 1 used a nonhormonal intrauterine device. Consequently, serum estradiol was measured prior to each trial to assure that there was no difference in its resting levels, as estrogen is one fluctuating endocrine factor that has been associated with sclerostin [23].

### 2.3. High Intensity Interval Exercise Trials

The two high intensity interval exercise trials (HIIR and HIIC) were randomly assigned in a crossover manner to each participant and scheduled one week apart. During each trial participants performed 8 intervals of 1 min cycling or running with 1 min recovery between intervals. The workload for the HIIR intervals was set using the maximum speed and incline achieved in the incremental running test. Similarly, the workload for the HIIC intervals was set using the maximum watts achieved in the incremental cycling test. To ensure high intensity, heart rate was recorded at the end of each interval. Mean heart rate and % of maximal heart rate for the 8 intervals were subsequently calculated for each participant. During both trials, participants' mean heart rate was >90% of maximum heart rate (93.2±4.7% for HIIR and 90.2±4.8% for HIIC). Borg rating of perceived exertion was recorded after each interval in both trials, with 19 being the mode value in both running and cycling trials.

### 2.4. Baseline Measurements

Height was measured with a stadiometer to the nearest 0.1 cm with no shoes. Waist and hip circumference were measured using a nonmetallic measuring tape and following standard procedures. Body composition was measured via air displacement plethysmography (BodPod; Life Measurement, Inc, USA) to get measures of body mass (kg), fat mass (kg), fat-free mass (kg) and percent body fat (%).

The Godin Shephard Leisure-Time Physical Activity Questionnaire was used to determine participants' habitual physical activity levels by calculating their leisure score index. A food frequency questionnaire (Block 2014.1_6Mo, Nutrition Quest, USA) was used to assess habitual nutrient intake.

### 2.5. Blood Collection and Biochemical Analysis

Venous blood samples were collected from the median cubital vein in the antecubital fossa of each participant using a standard venipuncture technique. In each high intensity interval exercise trial, approximately 10 ml of whole blood was collected from each participant at each time point (pre-exercise, 5 min, 1h, 24h, and 48h after exercise) for a total of 10 blood draws per participant. All blood samples sat for 30 min at room temperature before being centrifuged at 3000xg and 4°C for 15 min in a benchtop centrifuge (Allegra ZIR centrifuge, Beckman Coulter, USA). Serum was then aliquoted into microcentrifuge tubes and stored at -80°C until analysis. Plasma was also used to measure hematocrit.

Serum levels of sclerostin, CTXI, PINP, and baseline estradiol were measured in duplicate using commercially available immunoassay (ELISA) kits (SCL, cat.# DSST00, R&D Systems, Inc., CTXI, cat.# E-EL-H0835, Elabscience, and PINP, cat. # E-EL-H0185, Elabscience) following the manual instructions. Our in-house inter- and intra-assay coefficients of variation (CV) for sclerostin, CTXI, and PINP were 8.2% and 3.7%, 10% and 11.6%, and 7.2% and 9.9%, respectively. Baseline (i.e., resting) serum estradiol was measured prior to each trial using one ELISA kit (Estradiol, cat.# KGE014, R&D Systems, Inc., intra-assay CV: 8.2%).

### 2.6. Corrections for Plasma Volume Changes

Exercise (especially high intensity) induces plasma volume changes that can affect the interpretation of the observed response of biochemical measurements in blood [24, 25]. Thus, changes in biomarkers' measured concentrations are routinely adjusted for changes in plasma volume [24, 26, 27]. In the present study, percent plasma volume change (%ΔPV) from pre- to post-exercise was calculated for each participant using the following formula of Van Beaumont [27]:

(1) %ΔPV=100100-Hematocrit.pre-exercise x 100Hematocrit.pre-exercise-Hematocrit.post-exerciseHematocrit.post-exercise

This formula has been used in previous exercise studies [28–30], including those examining the effect of exercise on bone turnover markers such as CTXI and BAP [28]. Hematocrit was measured in triplicate by the same investigator, using microhematocrit tubes with heparin (VWR, USA) for each blood sample, and were separated using an international microcapillary centrifuge (model MB, International equipment company Needham, USA). Serum sclerostin, CTXI, and PINP levels at 5min, 1h, 24h, and 48h after exercise in both trials were corrected for plasma volume changes using the formula: 100 + %ΔPV/100. The mean % change in plasma volume was highest at 5 min after exercise ranging between -4.21% (in HIIR) and -6.34% (in HIIC).

### 2.7. Statistical Analysis

From a total of 200 blood samples collected in this study (20 participants x 10 sampling times), there was one missing sample due to one participant's absence from the 48h after HIIC blood draw. In addition, there were 3 missing values for CTXI because they were below the detection limit of the biochemical assay. Missing values were replaced with the mean value at the corresponding timepoint. Data were then assessed for normality using the Shapiro Wilk test, z-scores for skewness and kurtosis and visual screening of histograms for symmetry. Nonparametric tests were used in cases of violations of normality, which was the case for CTXI and PINP.

Paired t-tests were used to examine potential differences in sclerostin and estradiol levels at baseline, prior to the HIIR and HIIC trials, and nonparametric Wilcoxon tests were used to examine potential differences at baseline in CTXI and PINP. A two-way repeated measures analysis of variance (RM-ANOVA) was used to examine time and exercise mode main effects, as well as time-by-exercise mode interactions, for sclerostin. In the case of a significant time or exercise mode main effect, post hoc pairwise comparisons were performed using paired t-tests with Bonferroni adjustment for multiple comparisons. For CTXI and PINP, we used Friedman nonparametric analysis of repeated measures to examine the time effect within each trial. In case of a significant time main effect, post hoc pairwise comparisons were performed using Wilcoxon nonparametric tests with Bonferroni adjustment for multiple comparisons. Spearman correlations were used to examine potential associations between the absolute levels and % changes in sclerostin and bone turnover markers.

Effect sizes (ES), including partial eta squared f (partial *η*^2^) for ANOVA and Cohen's d (mean difference/standard deviation pretest) for significant pairwise comparisons were also calculated [31]. ES were then interpreted based on the Cohen criteria: 0.01=small, 0.06=moderate, 0.14=large effect for partial *η*^2^, and 0.2=small, 0.5=medium, and 0.8=large effect for Cohen's d [32]. Statistical significance was set at alpha level of 0.05 and performed using IBM SPSS Statistics 24 (SPSS Inc., Chicago, IL, USA).

## 3. Results

### 3.1. Baseline Characteristics

Participants' average percent body fat was within the normal range (Table 1). Participants had higher daily protein intakes than the 0.8 g/kg/day recommended dietary allowance (RDA), and calcium intake was slightly below the 1000 mg/day RDA (Table 1). Participants had similar baseline (i.e., resting) estradiol levels prior to the two high intensity interval exercise trials (99.8 ± 16.1 versus 93.3 ± 10.7, HIIR versus HIIC, respectively; p=0.47). Likewise, there were no differences at baseline between the two exercise trials in any of the other biomarkers (sclerostin, CTXI, and PINP).

### 3.2. Exercise Response (HIIR versus HIIC)

**Figure 2:**
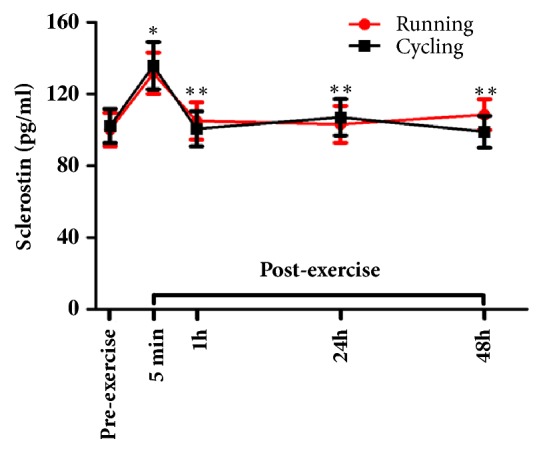
Serum concentrations of sclerostin (mean±SEM) before and after exercise in both high intensity interval exercise trials (running versus cycling). *∗* denotes significant difference from baseline to 5 min after exercise, *∗∗* denotes significant differences from 5 min to 1h and 24h, as well as between 5 min and 48h after exercise (paired t-test with Bonferroni adjustment, p<0.001).

There was a significant main time effect for sclerostin (F=18.20, p<0.001, partial *η*^2^ = 0.49), but no significant main exercise mode effect or exercise mode-by-time interaction. Post hoc pairwise comparisons showed that sclerostin levels increased significantly from pre-exercise to 5 min after exercise (p<0.001) and returned to near baseline levels 1h after exercise in both trials (Figure 2). The effect size (Cohen's d) for the change in sclerostin from pre-exercise to 5 min after exercise was large (0.8) for both trials (HIIR and HIIC). There was also no significant difference in sclerostin levels between HIIR and HIIC at any time point.

**Figure 3:**
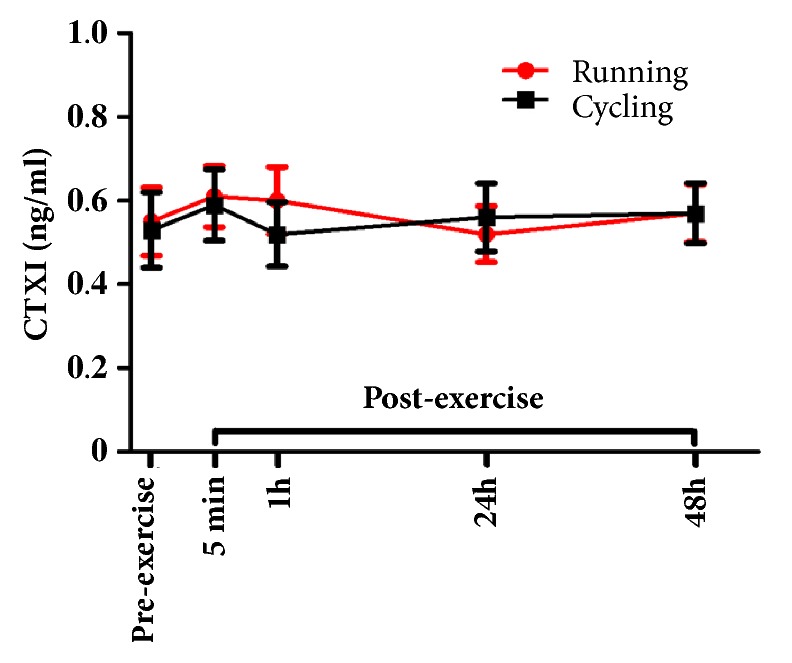
Serum concentrations of cross linked telopeptide of type I collagen (CTXI; mean±SEM) before and after exercise in both high intensity interval exercise trials (running versus cycling).

**Figure 4:**
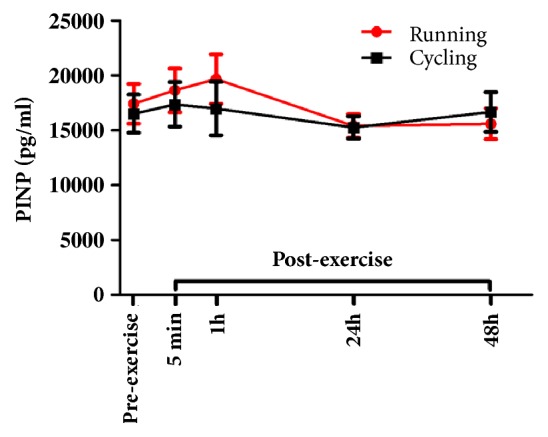
Serum concentrations of procollagen type I amino-terminal propeptide (PINP; mean±SEM) before and after exercise in both high intensity interval exercise trials (running versus cycling).

Friedman nonparametric analysis for repeated measures showed no significant main time effect for CTXI in either trial (Figure 3). A significant main time effect for PINP was found in HIIR (p=0.02), but not in HIIC. However, although PINP levels seemed to progressively increase 5 min and 1h following HIIR, decreasing 24h later, none of the post hoc pairwise comparisons reached statistical significance (Figure 4). In addition, there were no significant differences in CTXI and PINP concentrations between the two trials at any time point.

No significant correlations were found between the sclerostin, CTXI and PINP levels at any time point, nor in the % changes from pre-exercise to 5 min after exercise.

## 4. Discussion

This is the first study comparing the response of sclerostin and bone turnover markers to high intensity impact exercise (running) versus high intensity no-impact exercise (cycling) in the same group of participants, using a crossover design. We provided evidence that, consistent with previous studies in humans [12, 14], sclerostin increases 5 min after exercise, and this increase is similar between both modes of exercise, suggesting that impact (or no-impact) does not mediate sclerostin's response to exercise. In contrast, the PINP and CTXI response following this type of high intensity interval exercise did not appear to correspond to the sclerostin response. Additionally, there was no correlation between sclerostin and PINP or CTXI values at any time, suggesting that the increase in circulating sclerostin following high intensity exercise, with or without impact (i.e., gravitational forces,), does not lead to changes in the selected bone turnover markers within the 48h period following high intensity exercise.

### 4.1. Sclerostin's Response to High Intensity Running and Cycling

Based on previous findings in humans [12, 14, 15], we hypothesized that sclerostin would increase following high intensity exercise, and that there would be a larger magnitude of response following impact exercise, as this modality applies strong muscle forces as well as gravitational forces. Indeed, one study found resting sclerostin to be higher in male athletes of weight-bearing sports than of nonweight-bearing sports, yet this was not the case in their female cohort [33]. In contrast, we observed a transient post-exercise increase in sclerostin, of the same magnitude in both impact and no-impact exercise trials. This acute increase in sclerostin after exercise is in agreement with previous human studies, albeit the increase in the present study was of a smaller magnitude than previously reported; ~51% in young males 5 min after plyometric exercise [12] and ~44% in young females immediately after a moderate intensity running trial [14]. The higher percent increase in post-exercise sclerostin reported in these two previous studies compared with what we found in the present study (33.5% in HIIC and 35% in HIIR), might be attributed to exercise-induced plasma volume changes as neither previous study accounted for such changes. While it is important to control for any plasma volume changes induced by exercise [24, 25], previous studies have not consistently done so. For this reason, and to compare our findings with these studies, we also calculated our percent increase in uncorrected levels of sclerostin. Indeed, without correcting for plasma volume changes, the increase in sclerostin in the present study was similar to previous studies (41% in HIIR and 42% in HIIC). Irrespective of the above variability, our findings suggest that exercise performed at a high intensity can produce a similar sclerostin response regardless of impact. As previously speculated, the increase in serum sclerostin after exercise is likely due to the release of previously synthesized sclerostin from osteocytes into the blood, rather than to an increase in sclerostin's gene expression in this short period of time [14]. It is possible that the exercise-induced increase in blood flow to the bone [34] facilitated the release of the previously synthesized sclerostin, resulting in the acute increase immediately after exercise. Accordingly, sclerostin concentrations 1h, 24h, and 48h following the exercise, which were similar to pre-exercise levels, point to the transient, potentially noncatabolic significance of the 5 min after exercise increase.

Intuitively, based on previous* in vitro* [10] and* in vivo* animal [11] findings, one would expect sclerostin to decrease following exercise. However, all the acute exercise studies in humans have reported a transient acute increase. This increase in circulating sclerostin after exercise remains perplexing, especially since, based on the existing literature, we do not yet know how circulating sclerostin levels relate to its expression in the bone. One explanation for the difference seen between human and animal studies may lie in the duration of the mechanical loading, as exercise trials last only between 30 and 60 min, which is less than what has been applied in both* in vitro *experiments [10] and* in vivo *animal studies [11]. Another explanation is that the catabolic response observed in these young women might be related to an acute, transient decrease in the systemic energy availability due to exercise, which would lead to a short-term inhibition of Wnt signaling in bone as well as in peripheral tissues. Other transient catabolic responses to exercise, related to muscle activity, physical stress, and inflammation, have also been reported in athletes in relation to sclerostin [15, 35] and in nonathletes in relation to bone formation and resorption markers [20]. For example, sclerostin increased consistently and markedly during a 3-week cycling race suggesting a link between increased muscle activity and increased bone catabolism induced by the physical stress in absence of impact [15]. These questions cannot be answered by the present study, but they are intriguing and warrant further study. Alternatively, the increase in circulating sclerostin may be related to the recently suggested endocrine role of osteocytes and osteocyte-derived factors in energy and glucose metabolism [36] and in beige adipogenesis [37].

### 4.2. The Bone Turnover Response to High Intensity Running and Cycling

The above suggestion of sclerostin's potential nonbone post-exercise role is supported by the absence of a parallel response in our selected bone turnover markers. Indeed, despite the elevated sclerostin 5 min after exercise there was no indication of a subsequent decrease in bone formation and/or an increase in bone resorption markers following either trial, suggesting that the increase in circulating sclerostin immediately following these exercise trials did not directly target the Wnt pathway. It is possible, however, that 48h may not be long enough to see an effect in these bone turnover markers. An alternative reason for not finding clear time and mode effects on bone turnover markers might be that exercise has cumulative effects on bone. These effects, which are possibly mediated* via* exercise-induced sclerostin changes, may be significant over the long term, i.e., following long-term exercise training. This potential long-term training effect of sclerostin on bone turnover markers is supported by the findings of a longitudinal study reporting significant decreases in sclerostin after 8 weeks of a physical activity intervention, which was accompanied by a significant increase in bone formation markers (osteocalcin, PINP, and BAP) in sedentary premenopausal women [38].

It is also interesting that the selected bone turnover markers did not respond to high intensity interval exercise irrespective of impact. CTXI did not change following either trial, nor was there any significant difference in CTXI levels between running and cycling at any time after exercise. Likewise, although running seemed to result in an overall time effect for PINP, which increased 5 min (12%) and 1h (19%) after exercise, none of the post hoc pairwise comparisons were statistically significant. One previous study also showed no significant changes and no significant mode effect in either formation (osteocalcin, BAP) or resorption (NTX) markers following moderate jogging or water aerobic exercise in young women [22]. In contrast, our lab has shown that a similar trial of high intensity interval cycling leads to a significant increase in the bone formation marker (BAP) 5 min after exercise, and a later significant decrease in bone resorption (NTX) 24h after exercise, in young men [20]. However, these previous studies did not account for plasma volume changes, so it is also possible that the post-exercise increases in bone turnover markers reported therein [20, 39] were due to potential exercise-induced hemoconcentration, rather than to exercise per se. The discrepancy in the results may also be due to sex differences or to the use of different markers in the previous studies.

### 4.3. Strengths and Limitations

The main strengths of this study are its crossover, within-subject design, which allows for a direct comparison between the effects of high-impact and no-impact exercise in the same individuals. This design also lessens the necessity to control for individual differences such as race, ethnicity, birth control methods, etc., thus increasing the applicability of the results. The consideration of post-exercise plasma volume changes also adds rigour to the experimental design. The interpretation of any differences between no-impact exercise and impact exercise is conditional upon establishing whether either exercise is effective or ineffective in eliciting a response beyond the effects of hemoconcentration. Another strength is that the study participants were a homogeneous group in terms of body composition. Thus, there were no confounding issues of excess adiposity. Furthermore, all participants were on birth control ensuring absence of pregnancy and controlling for menstrual cycle related differences, and they had similar baseline estrogen concentrations in both trials. Finally, all testing occurred in the morning, following a consistent, standardized breakfast, thus avoiding any potential influence of diurnal variations or nutritional status.

The main limitation of this study is the absence of a non exercise control trial performed by all participants as part of the original experimental design. However, the consequent addition of a nonexercise control trial in the latter subset of participants confirmed that sclerostin resting levels can be considered stable during the morning hours. An intrinsic limitation of this study is that sclerostin was measured in serum and not at a cellular level (i.e., gene transcription or translation), which limits our mechanistic understanding of circulating sclerostin's connection to its expression in the osteocytes. In addition, these results must be interpreted with caution as it is difficult to extrapolate from one, acute exercise bout, how the body adapts to chronic loading (i.e., training).

### 4.4. Conclusion

High intensity interval exercise stimulates a response in circulating sclerostin immediately after exercise, regardless of the effect of gravitational loading (impact versus no-impact). Furthermore, the increase in circulating sclerostin following high intensity interval exercise was not accompanied by a subsequent decrease in bone formation nor an increase in bone resorption. Therefore, its role, up to 48 hours after high intensity exercise, remains unclear and must be further investigated.

## Figures and Tables

**Table 1:** Participant characteristics (n=20).

**Variable**	**Mean±SD**
Age (years)	22.5 ± 2.7
Height (cm)	156 ± 37
Body mass (kg)	58.9 ± 9.1
Body fat (%)	27.0 ± 7.0
Body fat-free mass (kg)	42.6 ± 5.2
Leisure score index (LSI)	57.9 ± 35.3
Energy intake (kcal·kg^−1^·day^−1^)	30.6 ± 7.9
Protein intake (grams·kg^−1^·day^−1^)	1.3 ± 0.7
Calcium intake (mg·day^−1^)	940.4 ± 304.0

## Data Availability

The blood and statistical data used to support the findings of this study are restricted by the Brock University Research Ethics Board in order to protect participant privacy. Data are available upon request from the corresponding author for researchers who meet the criteria for access to confidential data
